# Optimized ^13^C Relaxation-Filtered Nuclear Magnetic Resonance: Harnessing Optimal Control Pulses and Ultra-High Magnetic Fields for Metalloprotein Structural Elucidation

**DOI:** 10.3390/ijms26083870

**Published:** 2025-04-19

**Authors:** Leonardo Querci, Liza Burgassi, Simone Ciofi-Baffoni, Marco Schiavina, Mario Piccioli

**Affiliations:** 1 Department of Chemistry ‘Ugo Schiff’ (DICUS), University of Florence, 50019 Sesto Fiorentino, Italy; 2 Magnetic Resonance Center (CERM), University of Florence, Via Luigi Sacconi 6, 50019 Sesto Fiorentino, Italy; 3 Consorzio Interuniversitario Risonanze Magnetiche Metallo Proteine (CIRMMP), 50019 Sesto Fiorentino, Italy

**Keywords:** paramagnetic NMR, pNMR, biomolecular NMR, ^13^C direct detection, superWEFT, inversion recovery, optimal control pulses, PRE, paramagnetism-based structural restraints

## Abstract

Ultra-high magnetic fields and high-sensitivity cryoprobes permit the achievement of a high S/N ratio in ^13^C detection experiments, thus making a ^13^C superWEFT (Super water eliminated Fourier transform) experiment feasible. ^13^C signals that are not visible using ^1^H observed heteronuclear experiments, nor with established 2D ^13^C direct detection experiments, become easily observable when a ^13^C relaxation-based filter is used. Within this frame, optimal control pulses (OC pulses) have been, for the first time, applied to paramagnetic systems. Although the duration of OC pulses competes with relaxation, their application to paramagnetic signals has been successfully tested. OC pulses are much more efficient with respect to the phase- and amplitude-modulated ones routinely used at lower fields while providing bandwidth excitation profiles that are sufficient to meet the need to cover up to an 80 ppm spectral region. On the other hand, when paramagnetic relaxation is shorter than the duration of OC pulses, the use of hard, rectangular pulses is, at the present state of the art, the best approach to minimize the loss of signal intensity.

## 1. Introduction

The use of tailored NMR approaches to study paramagnetic systems dates back to about 40 years ago, when the evolution of NMR instrumentation allowed the design of many multi-pulse, fast recycling mono- and bi-dimensional experiments, tailored for the detection of fast relaxing and/or far-shifted ^1^H signals [1,2,3,4]. A very successful breakthrough was the optimization of an inversion recovery experiment, called superWEFT [5]. Although the acronym refers to an experiment optimized for the elimination of the strong water signal [6], this approach has been primarily adopted by the NMR community working on paramagnetic systems to suppress slow relaxing signals and enhance fast relaxing ones [7,8,9,10,11]. Later on, superWEFT was used as a longitudinal relaxation-based filter, applied prior to many homonuclear or heteronuclear experiments, to observe ^1^H-^1^H or ^1^H-^15^N or ^1^H-^13^C connectivities emerging from the crowded diamagnetic envelope of 2D spectra [12,13,14,15,16,17]. The development of ^13^C direct detection approaches [18], which rely on the possibility of detecting heteronuclear spins with highly sensitive and dedicated NMR probes [19,20,21,22], has represented a milestone in biomolecular NMR [23,24,25,26,27,28]. The direct excitation and observation of these nuclei allow us to use “protonless” coherence transfer pathways, which are particularly efficient in paramagnetic systems, where the dominant contributions to relaxation are γ^2^ dependent [29,30,31,32]. Under these circumstances, direct detection approaches decrease the blind sphere around the paramagnetic center, where signals are unobserved due to paramagnetic relaxation enhancement [33,34], adding a precious brick to the wall of NMR applications in biological inorganic chemistry [35,36,37,38].

In this frame, we show that a ^13^C superWEFT experiment, which identifies ^13^C signals that are not visible with ^1^H-^13^C experiments nor with established 2D ^13^C direct detected experiments [39], represents a precious and helpful piece of information. Essentially, this very simple experiment has become feasible thanks to the availability of ultra-high magnetic fields [40,41,42,43] that allow us to achieve a high S/N ratio in ^13^C detection experiments [44,45,46] and to expand the spectral region expressed in Hz, thus providing substantial resolution improvements.

The system that we used to set up the experiment is the human mitochondrial ferredoxin protein, FDX2, containing a [Fe_2_S_2_] cluster available in two oxidation states [Fe_2_S_2_]^2+/+^. The protein, identified in 2010 [47], is highly homologous to FDX1, which is also a [Fe_2_S_2_] mitochondrial protein. The X-ray structures of both these proteins in the proximity of the cluster are almost superimposable, a consideration that opened a debate concerning their functional specificity [48,49,50]. A structural and functional characterization of the active site at the atomic level helps to address differences between the two enzymes. This aspect becomes particularly important considering the recent data that showed that FDX2 has three different electron partner acceptors, all of which recognize the cluster binding region of FDX2 [51,52,53]. Therefore, an atomic-resolution tool able to map these FDX2 interactions is fundamental for defining the route of the electron transfer pathways. NMR spectroscopy exploiting ^13^C carbon spins affected by the paramagnetic interaction is an excellent option to obtain atomic-level information as close as possible to the metal binding site. Ultra-high magnetic fields, while increasing signal dispersion in the frequency domain (expressed in Hz), pose a challenge regarding the excitation of all the signals of interest. Rectangular pulses might not be the best choice for signal excitation and/or inversion anymore. However, amplitude- and phase-modulated pulses are bound to fail at such ultra-high fields. We will show here how the combination of high magnetic fields and optimal control pulses overcome the above limitations, providing broad inversion profiles that allow us to perform inversion recovery as well as more sophisticated experiments at 28.2 T over large spectral windows.

## 2. Results

### 2.1. ^13^C SuperWEFT Experiments Reveal Signals Not Visible with Other Experiments

**Figure 1:**
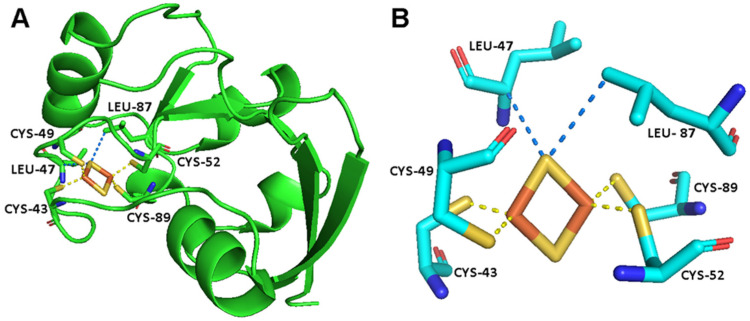
(**A**) The crystal structure of FDX2 [54]. (**B**) The metal binding site of FDX2, showing the [Fe_2_S_2_]^2+^ cluster, the iron bond residues and a few additional residues that are in electronic contact with the cluster [58].

**Figure 2:**
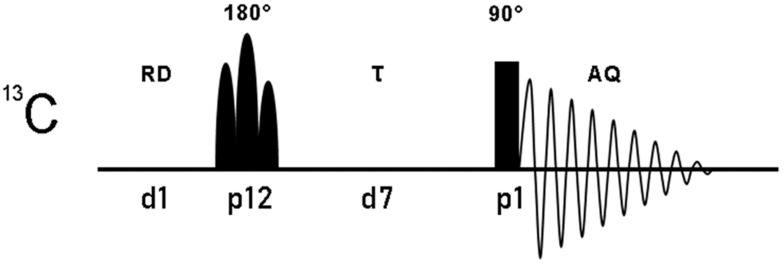
Pulse sequence of the one-dimensional ^13^C superWEFT experiment. ^13^C magnetization, inverted with a selectivity that depends on the nature and duration of the 180° pulse, is subjected to longitudinal relaxation during the delay τ and then acquired with a non-selective 90°. The overall recycle delay is given by the sum of the acquisition time (AQ) and the recycle delay (RD). Below the pulse sequence scheme, pulses and delays are reported according to the standard code of Bruker spectrometers language.

The environment of the [Fe_2_S_2_] cluster in FDX2 and the four cysteine residues bound to the cluster are shown in Figure 1 [54]. The superWEFT experiment, shown in Figure 2, can be used to identify signals that experience a nuclear spin relaxation enhancement due to the hyperfine interaction. The carbonyl region of the ^13^C spectrum is the benchmark to test the approach. The carbonyl spins of the Fe^3+^-bound cysteines are four bonds apart from Fe^3+^, and, therefore, no contact contributions to the carbonyl chemical shifts are expected [45]. However, there are also other C’ atoms belonging to the cluster binding loop, such as Gly 41, Ala 42, Ala 45 and Ala 48, that have their C’ atoms less than 5 Å away from the Fe^3+^, close enough to experience significant paramagnetic relaxation enhancement, as described by the Solomon–Bloembergen equation, which explains the electron–nucleus spins dipole–dipole relaxation [55,56,57]. Therefore, one can expect that, within the narrow spectral region where carbonyl signals usually resonate, a few signals experience paramagnetic relaxation enhancement and no paramagnetic shift. 

Under these circumstances, a hard and rectangular π pulse is sufficient to provide the inversion of all carbonyl spins. In order to make the experiment fast and effective, the use of an overall recycle delay (AQ+RD, in Figure 2), much shorter than the relaxation times of slow relaxing diamagnetic signals, has to be employed. This will cause a significant suppression of the intensity of slow relaxing signals, thus enhancing broad and fast relaxing ones. After the π inversion pulse, all signals are subjected to T_1_ relaxation during the delay τ, which acts as a longitudinal relaxation-based filter.

**Figure 3:**
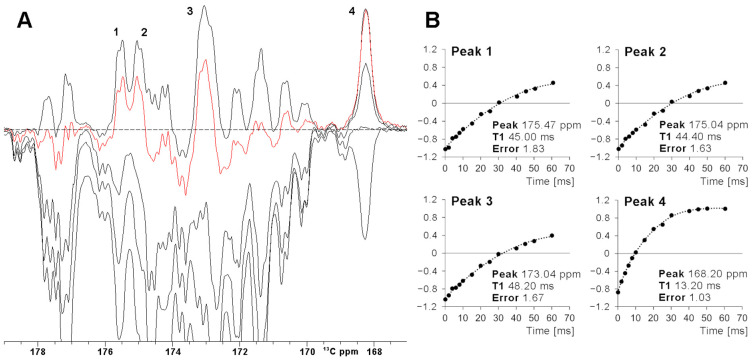
(**A**) SuperWEFT ^13^C experiments on the carbonyl carbon region of oxidized [Fe_2_S_2_] FDX2. The series was collected using inter-pulse delays from 100 us to 70 ms. The red trace was recorded with an inter-pulse delay equal to 45 ms. Four signals (1–4), relaxing faster than average, are unambiguously identified. (**B**) Signal intensities for peaks 1–4 vs the inter-pulse delay τ of the superWEFT experiment. In the case of signals 1–3, the magnetization is not completely recovered to the equilibrium along the z-axis during the recycle delay, and therefore, the time constants represent lower-limit estimates of the effective T_1_ values. In the case of signal 4, the recovery to the equilibrium is almost completed, and therefore, a reliable longitudinal relaxation time can be measured.

The efficiency of a superWEFT experiment essentially depends on the ratio between the recovery delay τ and the overall recycle delay. For a τ/(AQ+RD) ratio ca. 1, an NMR spectrum where all signals are positive is expected. At decreasing τ values, the inversion recovery gives rise, after the π/2 reading pulse, to positive signal intensity for fast relaxing signals and negative/near zero signal intensity for slow relaxing ones. This is evident in Figure 3A, where a superWEFT experiment was recorded initially with a recycle delay of 110 ms and a τ delay of 70 ms and then repeated at shorter τ delays. The series of experiments show that, for a 45 ms τ delay, only four signals (labeled 1–4 in Figure 3) remain positive, with unaffected or only minimally affected intensity. The fitting of the inversion recovery profile of these four signals permits us to precisely quantify the T_1_ of signal 4 at 13.4 ms and to obtain for the T_1_ values of signals 1–3 lower-limit values of 50 ms (signals 1–2) and 60 ms (signal 3). Consistently, the X-ray structure of FDX2 shows that Cys 49 C’ is the only carbonyl atom at 4.1 Å from the Fe^3+^, with the metal-to-carbon distance for the other C’ spins being ≥ 4.4 Å. The choice of the total recycle delay (AQ + RD) of superWEFT defines the range of T_1_ values that can be quantitatively measured with this experiment. Signals with 5∙T_1_ < RD are measured under “almost” steady state conditions, and, therefore, the rate constant of the inversion recovery profile will give the longitudinal relaxation times. On the other hand, signals that relax slower than the above limit will be partly saturated during the superWEFT, such as in the cases of signals 1–3 in Figure 3. Consequently, the rate constant obtained by the fitting is only a lower-limit estimate of the effective T_1_ values of the signals, as shown in Figure 3B.

### 2.2. Optimal Control Pulses Are Useful for Paramagnetic Systems

**Figure 4:**
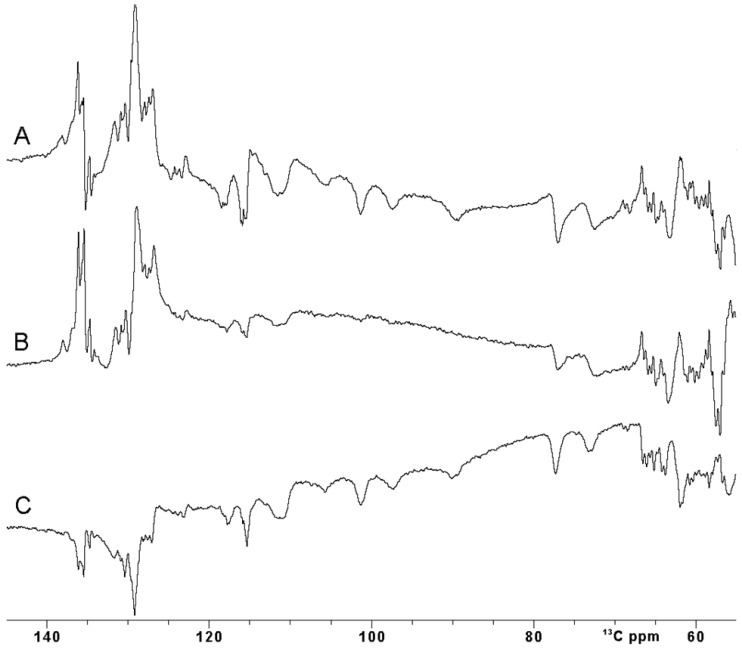
Comparison between ^13^C IR experiment performed with different inversion π pulses on oxidized [Fe_2_S_2_] FDX2. (**A**) A rectangular (Squa.100) pulse, (**B**) shaped (Q3.1000) pulse and (**C**) optimal control (SURBOP180) pulse [59,60]. The inter-pulse delay was 20 us. All the spectra were properly phased using both zero and first-order phase correction. Baseline correction was not applied.

Unfortunately, the use of rectangular hard pulses at very high fields has intrinsic limitations: a 26 µs 180° pulse, as used in this work, provides an efficient inversion over a +/− 16 ppm spectral region at 28.2 T for carbon spins. This is sufficient for the narrow carbonyl region but inadequate to study a ^13^C spectrum where signals are spread over a larger spectral window. Our case system is actually an excellent test to tackle this problem: C^β^ and C^α^ atoms of the four Fe^3+^-bound cysteine residues are only two or three σ bonds apart from the Fe^3+^ ions, and they experience up to 80 ppm of hyperfine contribution to their shift and, indeed, they are spread in the 140–60 ^13^C ppm region [58]. Figure 4A shows the inversion obtained using a rectangular pulse of 25 µs, with a carrier optimized to observe ^13^C paramagnetically shifted signals. As expected, a proper inversion is achieved only for signals in the 80–110 ppm region, and a non-homogenous inversion profile is observed outside this narrow spectral region.

Amplitude- and phase-modulated pulses, commonly known as shaped pulses, were introduced a long time ago for triple resonance experiments to address the need for uniform inversion profiles over a specific spectral region [59,61,62]. If the spectral region to be covered is huge, as in the case of the paramagnetic resonances, shaped pulses must be shortened to ensure effective irradiation. However, this shortening comes at the cost of increased power. At ultra-high fields when using cryoprobes, this power increase can exceed the maximum tolerable peak power of the NMR probe-head, making the use of such pulses unfeasible, particularly in samples with high ionic strength [63,64,65]. Furthermore, due to their pulse generation approach, even when the power remains within acceptable limits, shaped pulses often fail to meet performance requirements for large spectral widths [59,66]. This results in incomplete irradiation of the desired spins, even for the diamagnetic spin systems. Additionally, these pulses do not compensate for relaxation phenomena occurring during excitation, nor for the J coupling and/or chemical shift evolution [67]. Nevertheless, it is worth trying to invert the signals in the 70 ppm region of interest, exploiting a Q3.1000 pulse 240 µs long and with a corresponding power of 65 W [61]. The spectrum in Figure 4B clearly shows that the inversion made with the Q3.1000 pulse is dramatically affected by the performance of such a pulse at 1.2 GHz of proton Larmor frequency. In our case, fostering the pulse to invert a wide range of ppm results in an inefficient inversion. 

Optimal control pulses should be more appropriate to match our needs and to provide complete signal inversion over a large spectral region. Optimal control pulses are indeed designed to provide the maximum desired perturbation possible, exploiting low RF power even for very large spectral widths [60,68,69,70,71,72,73].

An optimal control SURBOP180 pulse, designed to cover an 80 ppm spectral width with a duration of 333 µs with a pulse power of 77 W, was used [60]. Of note, this OC inversion pulse, covering 24 kHz around the frequency carrier, is designed to exploit an rf-amplitude of only 15 kHz. The inversion experiment recorded with OC pulses (Figure 4C) definitely outplays the performance of the Q3.1000 shape inversion pulse in efficiently irradiating the desired spin systems. Even though OC pulses have a designed duration longer than the Q3.1000 inversion pulse previously described, their performance is comparable with the one obtained with a rectangular pulse.

**Figure 5:**
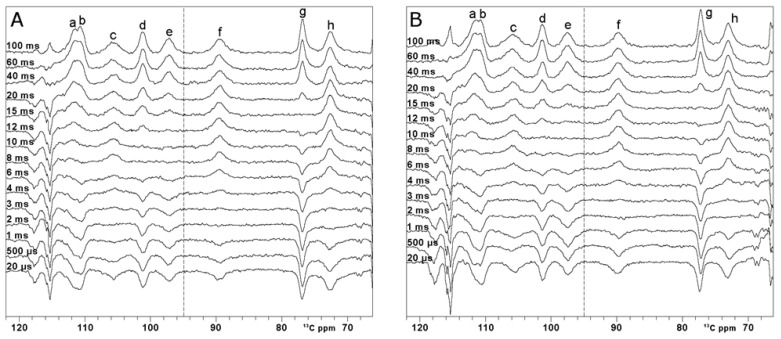
^13^C inversion recovery experiments—on oxidized [Fe_2_S_2_] FDX2. Series performed with variable inter-pulse delays from 100 ms to 20 µs, recorded using optimal control (**A**) and rectangular pulses (**B**) for the inversion. O1p = 95 ppm, AQ+RD = 100 ms. Within each series of inversion recovery experiments, all spectra were processed by applying the same first-order phase and polynomial baseline correction. Letters *a-h* refer to the different spin systems discussed in the text.

The applied SURBOP pulse is not designed to compensate for the relaxation mechanism during the pulse itself [74,75,76]. However, as reported in Figure 5A-B, which show the spectra obtained after manual polynomial baseline correction, the results obtained with OC pulses show a negligible effect of the relaxation process for the narrowest peaks (i.e., g and b having line widths of 257 Hz and 273 Hz, respectively), while the effect on the broader peaks results in a decrease in intensity of up to 50% (i.e., c and f having line widths of 552 Hz and 429 Hz, respectively).

These data suggest that signal relaxation, during the application of inversion pulses, is dominated by transverse relaxation rates. In metalloproteins and in paramagnetic molecules, transverse (and even longitudinal) relaxation times are often below 1 ms. The dominant mechanism contributing to transverse relaxation arises from the non-negligible Fermi contact relaxation [77,78]. In FDX2, most of the unpaired spin density is transferred on sulfide ions and then transferred, mainly via spin polarization mechanisms, not only to the C^α^ and C^β^ of Fe^3+^-bound cysteines, but also to aliphatic carbon atoms of neighboring residues, close enough to the sulfide ions of the cluster to sense a transfer of unpaired spin density via a Fe^3+^-S-H-C^ali^ path [15,58].

**Figure 6:**
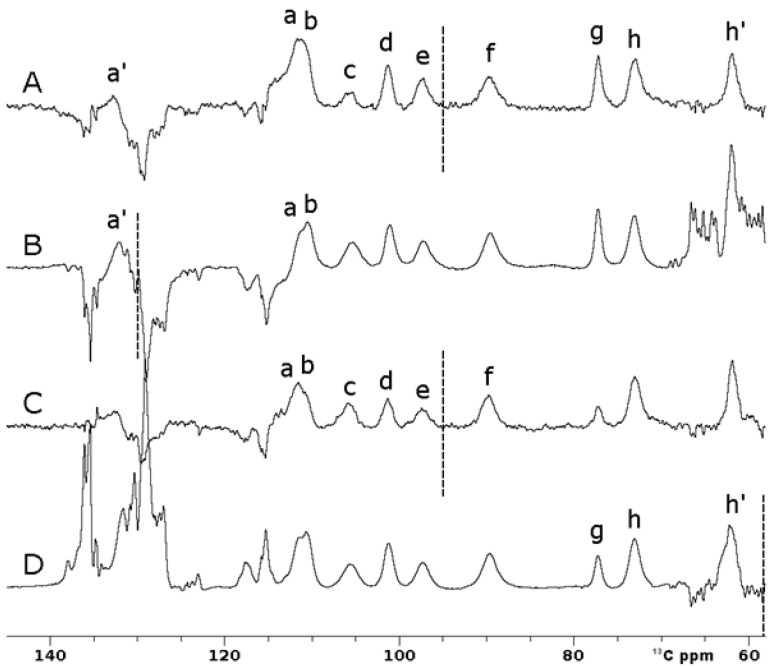
Comparison between ^13^C IR experiments on oxidized [Fe_2_S_2_] FDX2 performed using 100 ms of recycle delay and 25 ms of τ delay. Optimal control (**A**) and rectangular pulses (**B**, **C**, **D**) were used for the inversion. (**B**-**D**) Traces were obtained using frequency carriers of 130 ppm (**B**), 95 ppm (**C**) and 58 ppm (**D**), respectively. Spectra A and C were recorded with 16,384 scans, while B and D were recorded with 32,768 scans. Letters *a-h* refer to the different spin systems discussed in the text.

Figure 6A shows that superWEFT experiments with an OC inversion pulse allowed us to record a single spectrum where ten hyperfine-shifted and fast relaxing signals (labeled in the figure as a’, a-h, h’), in the range of 140–60 ^13^C ppm, can be unambiguously identified. Due to the inversion profile of rectangular inversion pulses, three independent experiments with these pulses were required to obtain the same information. The three spectra shown in Figure 6B, Figure Figure 6C and Figure Figure 6D, respectively, were recorded with the same experiment, where the carrier frequency was set to 130 ppm, 95 ppm and 58 ppm, respectively. The narrow bandwidth excitation of the π rectangular pulse is such that signal a’ can be observed only in Figure 6B; in Figure 6C, signals c–f can be observed with full intensity, while signal g is only partially inverted; finally, the upfield signal h’ clearly emerges only in Figure 6D. Signals a and b are slightly outside the excitation window in both 6B and 6C spectra, but the inversion was evident in both experiments. The superWEFT, based on the differential longitudinal relaxation rate between paramagnetic and diamagnetic signals, operates reliably only when signals are completely inverted. In the spectra obtained with rectangular inversion pulses, this condition was matched only for the narrow spectral region around the frequency carrier, i.e., 146–114 ppm in 6B, and 111–79 ppm and 74–42 ppm in 6D.

### 2.3. Inversion Recovery Curves Obtained with and Without OC Pulses

The paramagnetic ^13^C spectrum shows that signals a’ and h’ are overlapped with diamagnetic signals, and therefore, their T_1_ values cannot be accurately measured [58]. On the other hand, signals a–h resonate in a region where they are not hampered by diamagnetic spectral crowding. At variance with the carbonyl situation, all these signals are affected by paramagnetic relaxation to a larger extent. Thus, one can use superWEFT to obtain the inversion recovery curve and quantitatively measure T_1_ values, provided that paramagnetic relaxation rates can be used as structural restraints. Complete inversion recovery series are shown in Figure 5. For comparison purposes, two inversion recovery series using OC inversion pulses and rectangular inversion pulses were performed. T_1_ values and line widths are summarized in Table 1. The errors on the measured T_1_ values are below 15%, except for the two fastest relaxing signals, c and f, which display a 20% error. Within the uncertainty, the two series prove the same values of T_1_. The only signal that provides a different T_1_ value in the two series is signal c, which is the broadest peak of the spectrum. As observed from the spectra recorded at a very short τ delay, the OC inversion pulse is less efficient than the rectangular pulse, with the restriction that the signal falls within the very narrow excitation region of the rectangular pulse. This shows that, although the OC pulses are substantially more robust in covering a wider spectral region than shaped pulses, relaxation during the pulse is not compensated. Moreover, the possibility to properly irradiate a 24 kHz bandwidth enabled us to properly measure the T_1_ values of all the paramagnetic signals in a single set of experiments, shortening the overall experimental time.

## 3. Discussion

In paramagnetic systems, longitudinal relaxation rates depend on the γ^2^ of the evolving nuclear spin, and therefore, low-γ nuclei allow us to access structural information at progressively shorter metal-to-nucleus distances [30,32,79]. Oxidized FDX2 is a challenging playground to test this approach: the sequence-specific assignment of the protein is almost complete [51]; however, the signal identification of cysteine, ^1^H and ^13^C signals is not possible with routine NMR experiments due to the extreme broadening of these signals caused by the proximity to the paramagnetic center. However, a ^13^C NMR direct detection SuperWEFT is useful to identify paramagnetic resonances within a complex signal envelope, as shown in the case of carbonyl spins overwhelmed by the sharp, slow relaxing signals from residues far from the paramagnetic center. Provided that the molecular structure is available, signal assignment can be performed on the basis of metal-to-atom distances predicted by nuclear relaxation [79], such as the carbonyl signal at 170.7 ppm, assigned here as Cys 49 C’. Furthermore, eight hyperfine-shifted ^13^C signals are expected for a four-cysteine coordination [80,81]. However, ^13^C superWEFT was used to identify, in addition to eight hyperfine-shifted well-isolated signals, two additional paramagnetic ^13^C signals, buried in a crowded envelope and, therefore, not visible without the help of a relaxation-based filter. These results contribute to exhaustively describing the first coordination sphere of the [Fe_2_S_2_]^2+^ center. Within this framework, the use of ultra-high magnetic fields is crucial. Sensitivity is the major issue of ^13^C detected NMR; at the current state of the art, even when ^13^C-optimized cryoprobes are used, the gain in sensitivity given by the B0 field allows de facto operation with NMR approaches that, until a few years ago, were considered feasible only for the highly sensitive ^1^H nucleus. The availability of relaxation times of ^13^C signals in the immediate proximity of the paramagnetic center, obtained via an IR ^13^C direct detection experiment, constitutes a useful tool to retrieve structural restraints for structural elucidation [44,82,83,84]. 

An additional strength of measuring R_1_ values at high fields is represented by the possibility of expanding the range of action of paramagnetic relaxation enhancements. The observed longitudinal relaxation rates arise from Equation (1):

R_1_^obs^ = R_1_^para^ + R_1_^dia^ (1)

where R_1_^dia^ and R_1_^para^ are, respectively, the sum of all diamagnetic and paramagnetic contributions to the longitudinal relaxation rate. In a slow-motion regime, as in the case of biological macromolecules, R_1_^dia^ values decrease when the external magnetic field is increased. R_1_^para^ is obtained by subtracting the diamagnetic contribution from the experimentally measured R_1_^obs^; therefore, smaller R_1_^dia^ values resulted in the observation of R_1_^para^ contributions down to 1 s^−1^, enabling us to enlarge the sphere of the protein structure around the cluster that can be investigated using paramagnetic relaxation enhancement (PRE).

## 4. Materials and Methods

### 4.1. Protein Expression and Purification and NMR Sample Preparation

DNA coding for WT FDX2 (69-186) with an N-terminal 6 xHis-tag and a TEV-cleavage site was cloned into pET29b(+) and transformed into E. coli BL21 (DE3). The construct excludes the mitochondrial targeting sequence and the initial 13 residues. The complete expression and purification protocol is provided elsewhere. The NMR protein buffer was 30 mM HEPES (Sigma-Aldrich^®^, Kawasaki, Japan) and 150 mM NaCl at pH 7.5. A screw-capped 5 mm NMR tube containing 500 μL of ^13^C-^15^N-labeled FDX2 and 50 μL of D_2_O (Sigma-Aldrich^®^) was prepared. The protein concentration used in the NMR experiments was between 400 and 1100 μM.

### 4.2. NMR Data Collection and Processing

^13^C NMR spectra of oxidized FDX2 were acquired on a Bruker Ascend NMR spectrometer operating at the 300 MHz ^13^C Larmor frequency. The instrument was equipped with a cryogenically cooled, inverse detection TXO probe-head, optimized for direct detection of ^13^C spins. Measurements were performed at 298 K, using freshly prepared sample of ^13^C-^15^N-labeled oxidized FDX2. Inversion recovery experiments were performed using different π inversion pulses. The rectangular pulse (Squa.100) length was optimized using a *zg490* experiment, for a final value of 26 μs with 127 W. The calibration of the Q3.1000 shaped pulse was made with Topspin integrated software STDISP 4.0 for a final value of 240 μs with 65 W. The Surbop180 inversion pulse was designed to cover a 24 kHz bandwidth using a length of 333 μs and a power of 77 W. Two series of inversion recovery experiments were acquired to sample T_1_ relaxation times of ^13^C hyperfine-shifted signals from oxidized FDX2. The inversion recovery series differ only in the type of pulse used for the inversion of the signals: Squa.100 and Surbop180. Relaxation delays for signal recovery were as follows: 100 ms, 60 ms, 40 ms, 20 ms, 15 ms, 12 ms, 10 ms, 8 ms, 6 ms, 4 ms, 3 ms, 2 ms, 1 ms, 500 μs and 20 us. A total of 8192 time points were collected for a spectral window of 194 ppm. The frequency offset was 95 ppm. The acquisition and recycle delays were 70 ms and 30 ms, respectively. A total of 16,384 transients were acquired for each experiment. Additionally, one-dimensional ^13^C NMR spectra were acquired using a Squa.100 inversion pulse, at different frequency offset values: 130 ppm, 95 ppm and 58 ppm. Finally, a series of inversion recovery experiments using a rectangular 180° inversion pulse (26 μs at 127 W) was implemented on the carbonyl region of the ^13^C spectrum. Acquisition and recycle delays were 70 ms and 40 ms, respectively. The frequency offset was 175 ppm. A total of 32,768 transients were collected. Relaxation delays for signal recovery were as follows: 60 ms, 45 ms, 20 ms, 10 ms and 100 μs. All FIDs were processed using a squared sine function, with an SSB of 2, followed by an exponential multiplication (LB) of 10 Hz. Longitudinal relaxation times were estimated using Topspin integrated software Dynamic Center, using a single exponential function.

## 5. Conclusions

When approaching ultra-high-field NMR spectroscopy, we face the problem of covering large spectral regions. This is especially true for ^13^C protein NMR, where signals are spread over 200 ppm and the direct excitation of the complete spectrum is not possible. We have shown here, for the first time, that optimal control pulses are also suitable to address the problem in paramagnetic systems, where relaxation during the pulse plays a crucial role. We have also shown that these pulses are much more efficient with respect to the phase- and amplitude-modulated ones routinely used at lower fields such as the Q-shaped pulses.

Rectangular pulses provide bandwidth excitation profiles that are not sufficient to meet our need to cover up to an 80 ppm spectral region. However, when relaxation rates are > 200 s^−1^, the duration of OC pulses severely competes with relaxation, and the use of hard, rectangular, pulses is, at the present state of the art, the best approach to minimize the loss of signal intensity. On the other hand, optimal control pulses offer the possibility to combine wide and selective large bandwidth inversion with good robustness with respect to transverse relaxation. This aspect is critical for the application of ultra-high magnetic fields to paramagnetic proteins. In turn, this will be important to foster the study of field-dependent effects arising from the hyperfine interaction between unpaired electrons and nuclear spins.

## Figures and Tables

**Table 1:** Chemical shift, atom-type assignments and T_1_ values, measured with squared and optimal control inversion pulses, are reported for the different protein signals identified here as a’–h.

ID	^13^C δ_ppm_	Squa.100 180° T_1_ (ms)	Err.	Optimal Control 180° T_1_ (ms)	Err.	Line Width (Hz)	C-Type ass.
a’	134.4	/	/	/	/		Cys-C^α^
a	114.6	9.17	1.31	9.80	1.45	400	Cys-C^α^
b	113.6	12.5	1.56	12.2	1.72	270	Cys-C^α^
c	107.9	3.81	0.78	2.52	0.53	550	Cys-C^β^
d	104	12.8	0.99	11.8	1.60	310	Cys-C^α^
e	100	14.3	1.72	12.6	2.09	420	L87 C^δ^
f	92.4	3.67	0.41	3.52	0.81	430	Cys-C^β^
g	80	18.8	1.66	20.2	1.16	260	L47 C^α^
h	76.1	3.75	0.45	3.72	0.34	340	Cys-C^β^
h’	64.4	/	/	/	/		Cys-C^β^
1	178.1	/	/	/	/		C’
2	177.5	/	/	/	/		C’
3	175.6	/	/	/	/		C’
4	170.7	13.4	0.49	/	/		Cys-49 C’

## Data Availability

Datasets generated during this study are available upon request to the corresponding author.

## Abbreviations

The following abbreviations are used in this manuscript:

MDPI	Multidisciplinary Digital Publishing Institute
S/N	Signal-to-Noise
OC	optimal control
NMR	Nuclear Magnetic Resonance
WEFT	Water-Eliminated Fourier Transform
Hz	Hertz
FDX2	Human Ferredoxin 2
FDX1	Adrenodoxin
AQ	acquisition delay
RD	recycle delay
RF	Radio Frequency
IR	inversion recovery
PRE	paramagnetic relaxation enhancement
